# Prognostic Value of a Ferroptosis-Related Gene Signature in Patients With Head and Neck Squamous Cell Carcinoma

**DOI:** 10.3389/fcell.2021.739011

**Published:** 2021-11-01

**Authors:** Dongsheng He, Shengyin Liao, Linlin Xiao, Lifang Cai, Mengxing You, Limei He, Weiming Huang

**Affiliations:** Department of Medical Oncology, The First Hospital of Putian, Teaching Hospital, Fujian Medical University, Putian, China

**Keywords:** HNSCC, ferroptosis, prognosis, nomogram, immunotherapy

## Abstract

**Background:** Ferroptosis is an iron-dependent programmed cell death (PCD) form that plays a crucial role in tumorigenesis and might affect the antitumor effect of radiotherapy and immunotherapy. This study aimed to investigate distinct ferroptosis-related genes, their prognostic value and their relationship with immunotherapy in patients with head and neck squamous cell carcinoma (HNSCC).

**Methods:** The differentially expressed ferroptosis-related genes in HNSCC were filtered based on multiple public databases. To avoid overfitting and improve clinical practicability, univariable, least absolute shrinkage and selection operator (LASSO) and multivariable Cox algorithms were performed to construct a prognostic risk model. Moreover, a nomogram was constructed to forecast individual prognosis. The differences in tumor mutational burden (TMB), immune infiltration and immune checkpoint genes in HNSCC patients with different prognoses were investigated. The correlation between drug sensitivity and the model was firstly analyzed by the Pearson method.

**Results:** Ten genes related to ferroptosis were screened to construct the prognostic risk model. Kaplan-Meier (K-M) analysis showed that the prognosis of HNSCC patients in the high-risk group was significantly lower than that in the low-risk group (*P* < 0.001), and the area under the curve (AUC) of the 1-, 3- and 5-year receiver operating characteristic (ROC) curve increased year by year (0.665, 0.743, and 0.755). The internal and external validation further verified the accuracy of the model. Then, a nomogram was build based on the reliable model. The C-index of the nomogram was superior to a previous study (0.752 vs. 0.640), and the AUC (0.729 vs. 0.597 at 1 year, 0.828 vs. 0.706 at 3 years and 0.853 vs. 0.645 at 5 years), calibration plot and decision curve analysis (DCA) also shown the satisfactory predictive capacity. Furthermore, the TMB was revealed to be positively correlated with the risk score in HNSCC patients (*R* = 0.14; *P* < 0.01). The differences in immune infiltration and immune checkpoint genes were significant (*P* < 0.05). Pearson analysis showed that the relationship between the model and the sensitivity to antitumor drugs was significant (*P* < 0.05).

**Conclusion:** Our findings identified potential novel therapeutic targets, providing further potential improvement in the individualized treatment of patients with HNSCC.

## Introduction

Head and neck cancer is the sixth most common malignancy that leads to considerable mortality, with >450,000 deaths reported worldwide in 2020 (International Agency for Research on Cancer, and World Health Organization, 2021). The incidence rate of head and neck squamous cell carcinoma (HNSCC), which is the most common type of head and neck cancer, is approximately 20 per 100000 people in the regions of South America, China, Europe, the Indian subcontinent, and among African Americans in the United States (Stenson, 2021). The well-known risk factors for HNSCC are chronic exposure to alcohol, smoking, different forms of chewing tobacco (such as betel palm), chronic oral trauma, and HPV infection (Gillison et al., 2015). Currently, surgical resection, radiotherapy, chemotherapy, targeted therapy, and immunotherapy have been developed to comprehensively treat HNSCC patients. However, the 5-year overall survival (OS) of HNSCC patients has remained at 50%, which has not significantly improved in the past decade (Torre et al., 2015; Bray et al., 2018). Moreover, mounting evidence highlights that the percentage of locoregional failure is approximately 40–50%, while distant failure is 20–30% in locoregionally advanced HNSCC patients (Samra et al., 2018; Muzaffar et al., 2021). Therefore, studies exploring novel therapeutic targets and developing novel prognostic models to identify patients with different prognoses are urgently required for HNSCC patients.

Iron is an important transition metal for maintaining the rapid proliferation and growth of cancer cells (Zhou et al., 2018). Moreover, iron participates in several important biological processes, such as oxygen transport, DNA synthesis, and ATP generation. However, excess intracellular iron accumulation can trigger reactive oxygen species (ROS), which cause lipid peroxidation and ferroptosis, a unique form of cell death (Bogdan et al., 2016; Fanzani and Poli, 2017). Ferroptosis is defined as an iron-dependent programmed cell death (PCD) that is dependent on ROS accumulation and lipid peroxidation, and the mechanism and morphology of PCD are distinct from other PCDs, such as autophagy, apoptosis, and necroptosis (Dixon et al., 2012; Stockwell et al., 2017). In recent decades, research on ferroptosis in tumors has rapidly increased, and this PCD has been indicated to be correlated with tumor origin, development, and treatment (Liang et al., 2019; Stockwell and Jiang, 2019). Ferroptosis regulatory genes, such as P53, DPP4, and GPX4, have been shown to be correlated with tumorigenesis and progression (Junttila and Evan, 2009; Liu et al., 2018; Enz et al., 2019). Moreover, ferroptosis has been suggested to regulate the sensitivity of tumor cells to radiotherapy and immunotherapy. Radiotherapy has been suggested to induce ferroptosis, which plays a crucial role in radiotherapy-mediated anticancer effects, and improve the sensitivity of tumor cells to ferroptosis inducers (Lang et al., 2019). Furthermore, interferon-gamma and T cells may sensitize tumor cells to ferroptosis (Wang et al., 2019). After treatment of tumor models with immune checkpoint inhibitors, ROS levels were significantly increased, while tumor size was significantly reduced (Mou et al., 2019). Radiotherapy and immunotherapy have been shown to promote lipid oxidation and ferroptosis in tumor cells owing to a synergistic effect (Lang et al., 2019). Nevertheless, the prognostic value and relationship of distinct ferroptosis-related genes with immunotherapy in HNSCC remain predominantly unknown.

In this study, we first integrated The Cancer Genome Atlas (TCGA), Genotype-Tissue Expression (GTEx), and ArrayExpress databases to construct and validate a novel prognostic risk model based on ferroptosis-related messenger RNA (mRNA). Moreover, internal and external validations were first used to assess the risk model accuracy compared with a previous study (He et al., 2021). To the best of our knowledge, the prognostic risk model and nomogram based on the model showed the best prognostic effect in the present study of risk models based on ferroptosis-related mRNA (He et al., 2021). In addition, the values of the model used to predict prognosis with other clinical parameters and immunotherapy of HNSCC patients were further explored. The correlation between the prognostic risk model and drug sensitivity in cancers was first analyzed.

## Materials and Methods

### Patients and Clinical Data Acquisition

In this study, mRNA sequencing data (fragments per kilobase million) of HNSCC patients were obtained from TCGA database^1^ (44 normal head and neck samples and 501 HNSCC samples). To further strengthen the reliability, the sequencing data of 55 salivary gland samples in the GTEx^2^ database were obtained using the UCSC Xena tool and integrated with HNSCC sequencing data from TCGA database (Cao et al., 2019). The integrated data were normalized and processed using the Limma R package (Ritchie et al., 2015). Moreover, the clinical data of HNSCC patients were downloaded from TCGA database.

### Acquisition of Differentially Expressed Ferroptosis-Related Genes in HNSCC Patients

The Limma package was used to screen the differentially expressed genes (DEGs) in HNSCC patients with a false discovery rate (FDR) <0.05, and fold change > 2 (Wu et al., 2020). Ferroptosis-related gene sets (driver, suppressor, and marker) were downloaded from FerrDb, the first manually curated database of ferroptosis regulators and markers and ferroptosis-disease associations. The database provides up-to-date and comprehensive ferroptosis-related genes (Zhou and Bao, 2020). Moreover, a ferroptosis-related gene set (WP_FERROPTOSIS) in the Molecular Signature Database v7.2 (MSigDB) was downloaded (Liberzon et al., 2015). The ferroptosis-related genes were identified after removing the overlapping genes. Differentially expressed ferroptosis-related genes were identified by intersecting ferroptosis-related genes with DEGs.

### Construction and Assessment of the Risk Score Prognostic Model

The differentially expressed ferroptosis-related genes in HNSCC patients were matched with the corresponding survival time and status (*n* = 498) after excluding missing data. HNSCC patients were then randomly separated into training and testing groups using the caret package at a ratio of 7:3 (Zhang et al., 2020). A univariate Cox regression algorithm was employed to screen for ferroptosis-related genes associated with OS in the training group (*P* < 0.05). The least absolute shrinkage and selection operator (LASSO) logistic regression algorithm was used to avoid overfitting. Finally, a multivariable Cox regression analysis was conducted to construct a prognostic risk score model. HNSCC patients were divided into high- and low-risk groups based on the median risk score as the cutoff value. The Kaplan-Meier (K-M) method and receiver operating characteristic (ROC) curves were used to validate model feasibility.

### Internal and External Validation of the Risk Score Prognostic Model

HNSCC patients in the testing cohort were divided into high- and low-risk groups based on the median risk score in the training cohort. The K-M method and ROC curve were used to test the feasibility of internal validation. Moreover, the prognostic capability of the risk score model was externally validated in the entire cohort and the ArrayExpress database^3^. The mRNA sequencing data and corresponding clinical information of HNSCC (*n* = 108) were obtained from the E-MTAB-8588 dataset in the ArrayExpress database. The risk score was calculated for HNSCC patients using the prognostic model in the training cohort, and the “sva” R package was utilized to eliminate different dataset biases (Leek et al., 2012).

### Functional Enrichment Analysis of the 10-Gene Signature

Gene Ontology (GO) analysis is a major bioinformatics tool for annotating genes and gene functions (Gaudet et al., 2017). The Kyoto Encyclopedia of Genes and Genomes (KEGG) is a collection of databases that contain information regarding genomes, biological pathways, diseases, and chemical substances (Kanehisa et al., 2017). The “clusterProfiler” R package was employed to explore the gene function and pathway of the 10 genes (Liu M. et al., 2021). Differences were considered statistically significant at FDR < 0.05.

### Construction and Assessment of the Nomogram for HNSCC Patients

To further assess the capability of the risk score prognostic model to be an independent prognostic factor, the risk score of HNSCC patients was integrated with the clinical parameters of HNSCC patients in the training group. The inclusion criteria for HNSCC patients were as follows: (1) pathological type was squamous cell carcinoma, (2) complete follow-up data, (3) received chemotherapy or radiotherapy, and (4) clear pathological stage. A univariate Cox regression algorithm was used to screen out the OS-related characteristics in HNSCC patients (*P* < 0.05), and multivariable Cox regression analyses were used to identify independent prognostic parameters. Subsequently, a nomogram was constructed to predict an individual’s OS based on the independent prognostic parameters, and the concordance index (C-index), ROC curve, calibration plot, and decision curve analysis (DCA) were used to determine the prognostic ability of the nomogram from multiple perspectives. The nomogram was further validated in the testing, entire, and external groups.

### Analysis of the Relationship Between the Immune Microenvironment and Risk Score Model in HNSCC Patients

The single nucleotide variant (SNV) data of HNSCC patients were obtained from TCGA database, and tumor mutational burden (TMB) was calculated for each HNSCC patient. The correlation analysis between risk score and TMB was conducted using Spearman’s algorithm, and the difference in TMB between the high- and low-risk groups was explored. Moreover, the “Cell Type Identification by Estimating Relative Subsets of RNA Transcripts (CIBERSORT)” deconvolution algorithm with 1,000 permutations was applied to quantify 22 tumor-infiltrating lymphocyte types in the microenvironment of high- and low-risk HNSCC patients (Becht et al., 2016). Statistical significance was set at *P* < 0.05.

### Exploration of Drug Sensitivity Based on the Prognostic Model

To explore the anticancer drugs targeted to the prognostic model, the sensitivity information of anticancer drugs approved by the United States Food and Drug Administration for use in the clinic, and RNA sequencing data in the NCI 60 platform were downloaded from the CellMiner database^4^ (Foy et al., 2017). The correlation between ferroptosis-related genes to construct a prognostic model and drug sensitivity in cancers was analyzed using Pearson analysis (Reinhold et al., 2019).

## Results

### Identification of Ferroptosis-Related Genes in HNSCC

**FIGURE 1:**
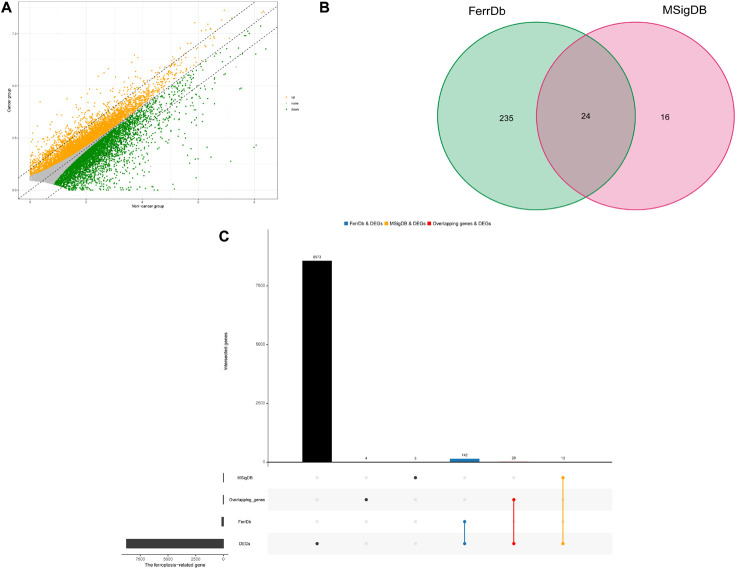
Identification of differential expressed ferroptosis-related genes in HNSCC. **(A)** Differential expressed genes (DEGs) of head and neck squamous cell carcinoma (HNSCC). **(B)** Venn diagram of ferroptosis-related gene sets. **(C)** The blue and orange bars represents the unique DEGs related to ferroptosis in HNSCC in the FerrDb and MSigDB, respectively, and the red bar represents the overlapping DEGs related to ferroptosis in HNSCC between the FerrDb and MSigDB.

A total of 8655 DEGs were screened out in HNSCC based on TCGA and GTEx databases (Figure 1A). After overlapping genes were filtered, 275 ferroptosis-related genes were screened based on the FerrDb and MSigDB (Figure 1B). Next, 175 differentially expressed ferroptosis-related genes in HNSCC were identified by intersecting the DEGs and ferroptosis-related genes (Figure 1C).

### Construction and Assessment of the Risk Score Prognostic Model

**FIGURE 2:**
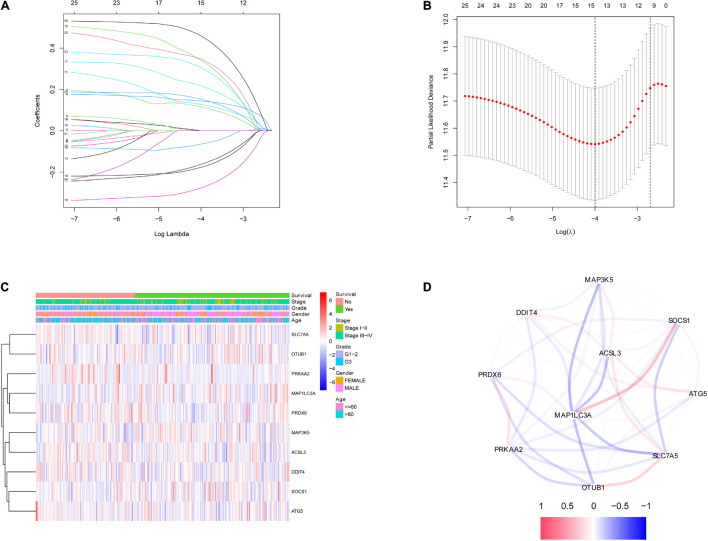
Identification of ferroptosis-related signatures by LASSO regression algorithm in HNSCC. **(A)** LASSO coefficient profiles of the 25 ferroptosis-related genes. **(B)** Cross-validation for tuning parameter selection in the proportional hazards model. **(C)** The heatmap of the 10 ferroptosis-related genes. **(D)** The correlation of the 10 ferroptosis-related genes.

**FIGURE 3:**
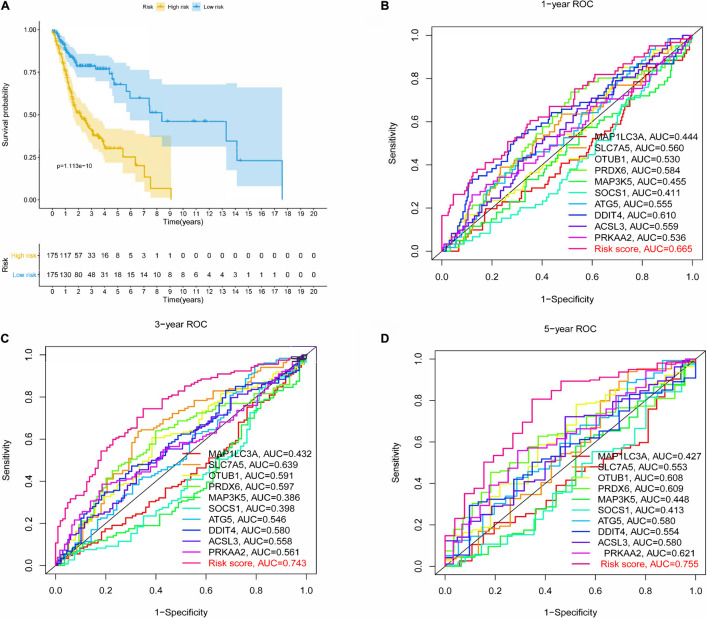
Kaplan-Meier (K-M) survival analysis and receiver operating characteristic (ROC) curves of risk prognostic model in HNSCC patients. **(A)** K-M survival analysis of risk prognostic model of HNSCC patients in training cohort. **(B–D)** ROC curves analysis of risk prognostic model of HNSCC patients at 1, 3, and 5 years in training cohort.

After the survival data were integrated with the expression profile of 175 ferroptosis-related genes, HNSCC patients were randomly divided into training (*n* = 350) and testing cohorts (*n* = 148) at a ratio of 7:3. Univariate Cox regression analysis was performed to screen 25 genes related to the OS of HNSCC patients in the training cohort (Supplementary Table 1). The 25 filtered genes were further included in the LASSO logistic regression algorithm to avoid overfitting (Figure 2A), and cross-validation was conducted, which filtered out 13 prognostic signatures (Figure 2B). Next, 10 gene signatures were screened to construct the following prognostic risk model by multivariable Cox regression analyses (Supplementary Figure 1): Risk score = (−0.191 × MAP1LC3A expression level) + (0.189 × SLC7A5 expression level) + (0.525 × OTUB1 expression level) + (0.399 × PRDX6 expression level) + (−0.374 × MAP3K5 expression level) + (−0.258 × SOCS1 expression level) + (0.555 × ATG5 expression level) + (0.227 × DDIT4 expression level) + (0.406 × ACSL3 expression level) + (0.602 × PRKAA2 expression level; Figure 2C). The correlation network of the 10 genes is shown in Figure 2D. Training group HNSCC patients were divided into high- (*n* = 175) and low-risk groups (*n* = 175) based on the median risk score. The OS in the low-risk group was significantly better than that in the high-risk group according to the K-M analysis (*P* < 0.001; Figure 3A). As shown in Figures 3B–D, the area under the curve (AUC) values for the ROC curves at 1, 3, and 5 years are 0.665, 0.743, and 0.755, respectively, increasing annually.

To further verify the predictive capability of the model in the training cohort, the testing cohort was used for internal validation, and the entire cohort and E-MTAB-8588 dataset were used for external validation. HNSCC patients in the testing cohort were divided into high- (*n* = 65) and low-risk (*n* = 83) groups based on the risk model in the training cohort. The OS of the two groups differed significantly in the K-M analysis (*P* < 0.01; Supplementary Figure 2A), and the AUC values at 1, 3, and 5 years were 0.648, 0.688, and 0.659, respectively (Supplementary Figures 2D–F). Similar to internal validation, external validation further strengthens model reliability during training. As shown in Supplementary Figures 2B,C,K-M analysis shows a significant difference between the high- and low-risk groups in the entire group (*P* < 0.001) and MTAB-8588 dataset (*P* = 0.03). The AUC values at 1, 3, and 5 years were 0.660, 0.718, and 0.713, respectively, for the entire cohort, and 0.632, 0.687, and 0.647, respectively, for the MTAB-8588 dataset (Supplementary Figures 2G–L). Overall, the accuracy increased as the sample size increased.

### Functional Enrichment Analysis of the 10 Ferroptosis-Related Genes

The most highly enriched functions of the 10 screened genes were cellular response to chemical stress, starvation, nutrient levels, and oxidative stress (Supplementary Figure 3A). Moreover, KEGG pathway analysis demonstrated that the 10 genes might participate in ferroptosis via autophagy or mTOR signaling pathways (Supplementary Figure 3B). The involvement of these potential pathways in ferroptosis has been previously reported (Liu et al., 2020; Yi et al., 2020).

### Construction and Assessment of the Nomogram for HNSCC Patients

**FIGURE 4:**
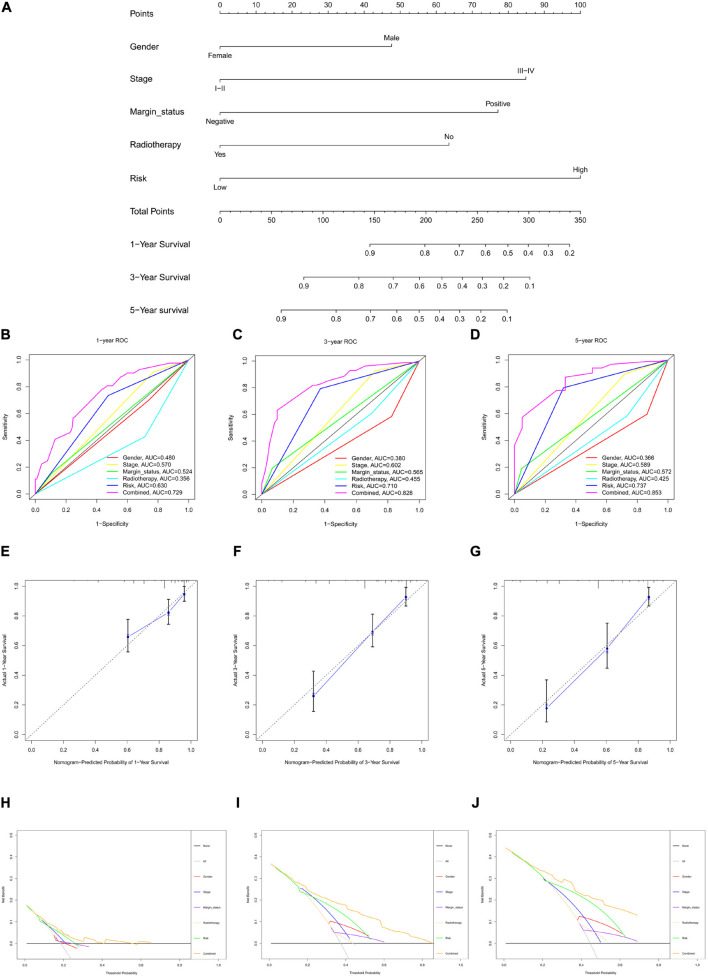
Nomogram to predict 1-, 3-, and 5-year overall survival (OS) and its validation in HNSCC patients. **(A)** Nomogram to predict 1-, 3-, and 5-year OS of HNSCC patients. **(B–D)** ROC curves to assess nomogram accuracy to predict 1-, 3-, and 5-year OS in HNSCC patients. **(E–G)** Calibration plot analysis to assess nomogram accuracy of to predict 1-, 3-, and 5-year OS in HNSCC patients. **(H–J)** Decision curve analysis to assess nomogram accuracy to predict 1-, 3-, and 5-year OS in HNSCC patients.

The risk score for each HNSCC patient in the training cohort was integrated with age, sex, grade, stage, margin status, radiotherapy, and chemotherapy (*n* = 243). Five clinical characteristics, namely sex, stage, margin status, radiotherapy, and risk, were found by univariate Cox regression analysis to be correlated with prognosis (Supplementary Figure 4A). A multivariable Cox algorithm was used to identify the five independent prognostic parameters of HNSCC patients (Supplementary Figure 4B). A nomogram with a satisfactory C-index (0.752) was constructed based on independent prognostic parameters (Figure 4A). Moreover, ROC curve analysis was employed to determine the nomogram accuracy for predicting the 1-, 3-, and 5-year OS of individuals, and the AUC values revealed optimal values (0.729, 0.828, and 0.853, respectively), increasing annually (Figures 4B–D). The actual curve was similar to the ideal curve in the calibration plot (Figures 4E–G). DCA further confirmed the creditability of the prognostic effect of the nomogram, and the risk and combined curves were superior to other factors in forecasting individual prognosis (Figures 4H–J).

To validate the prognostic value of the nomogram, the risk score of the testing cohort was integrated with the corresponding clinical information for internal validation, and the entire cohort and E-MTAB-8588 dataset were integrated with the corresponding clinical parameters for external validation. In the testing cohort, the C-index was 0.629, and the AUC values at 1, 3, and 5 years were 0.645, 0.693, and 0.717, respectively, increasing annually (Supplementary Figures 5A–C). In the entire cohort (*n* = 361), the C-index was 0.721, and the AUC values at 1, 3, and 5 years were 0.738, 0.770, and 0.820, respectively, increasing annually (Supplementary Figures 5D–F). In the E-MTAB-8588 dataset (*n* = 107), the C-index was 0.600, and the AUC values at 1, 3, and 5 years were 0.604, 0.641, and 0.611, respectively, when margin status data were missing (Supplementary Figures 5G–I). Together, the internal and external validations strengthened the creditability of the nomogram. Internal and external validation suggested that the prognostic effect of the risk score model and the model-based nomogram reliable. Moreover, the risk score differed significantly in the T stage, N stage, lymphovascular invasion, and perineural invasion (Supplementary Figure 6).

### Analysis of the Relationship Between the Immune Microenvironment and Risk Score Model in HNSCC Patients

**FIGURE 5:**
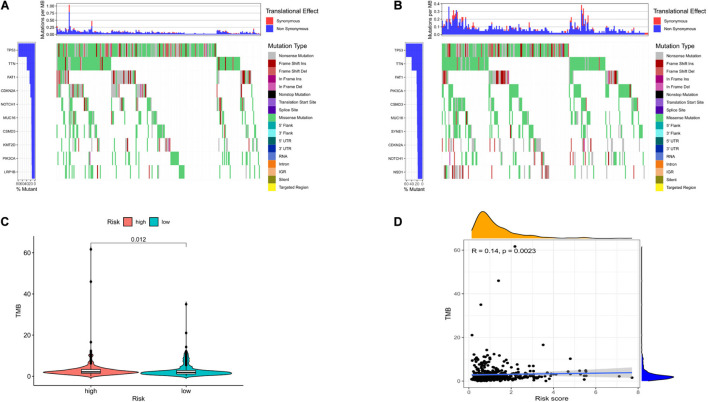
The differences of tumor mutational burden (TMB) in high- and low-risk HNSCC patients. **(A)** The TMB in high-risk HNSCC patients. **(B)** The TMB in low-risk HNSCC patients. **(C)** The difference of TMB was significant in HNSCC patients with different risk. **(D)** The TMB was positively correlated with the risk score in HNSCC patients.

To investigate the relationship between risk model-related ferroptosis and TMB, the risk score was integrated with SNV in HNSCC patients, and the TMB was significantly different in the high- and low-risk groups (Figures 5A–C). Moreover, the correlation analysis indicated that TMB was positively correlated with the risk score (*R* = 0.14, *P* < 0.01; Figure 5D). However, a high TMB might fail to predict the immune checkpoint blockade response across all cancer types.

**FIGURE 6:**
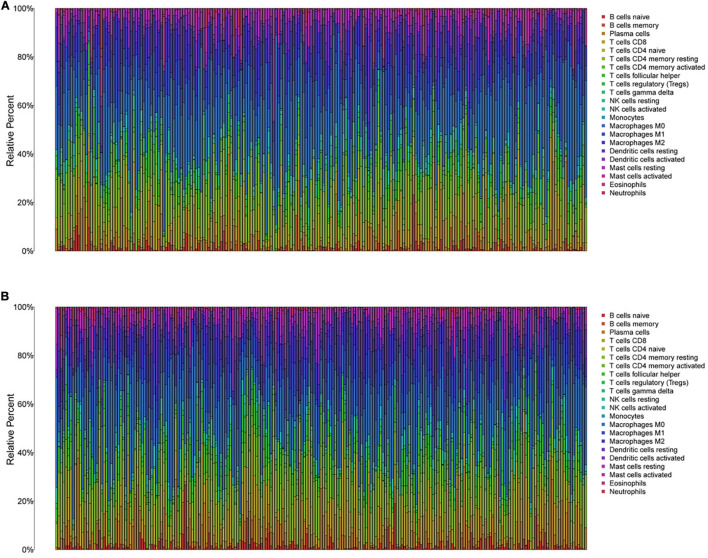
The immune infiltration of 22 immune cell types in high- and low-risk HNSCC patients. **(A)** The immune infiltration of 22 immune cell types in high-risk HNSCC patients. **(B)** The immune infiltration of 22 immune cell types in low-risk HNSCC patients.

To further explore the individual immune microenvironment and develop individualized treatment, immune infiltration and immune checkpoint genes in high- and low-risk HNSCC patients were further investigated (Figures 6A,B). Compared to the high-risk patients, the low-risk HNSCC patients had higher marker expression of naive B cells (*P* < 0.001), plasma cells (*P* < 0.05), CD8 T cells (*P* < 0.001), follicular helper T cells (*P* < 0.01), regulatory T cells (Tregs, *P* < 0.001), gamma delta T cells (*P* < 0.01), resting mast cells (*P* < 0.05), and neutrophils (*P* < 0.001), however, lower marker expression of resting NK cells (*P* < 0.01), M0 macrophages (*P* < 0.05), M2 macrophages *P* < 0.05), activated mast cells (*P* < 0.05), and eosinophils (*P* < 0.01; Supplementary Figure 7). Moreover, differences in immune checkpoint genes in high- and low-risk HNSCC patients were identified. The expression levels of IL10, CTLA4, PD1, TNFRSF14, BTLA, LGALS9, TIGIT, LAG3, EDNRB, and IDO1 were higher in the low-risk group than those in the high-risk group. Contrastingly, the high-risk group had higher MICA and MICB expressions compared to that of the low-risk group (Supplementary Figure 8). The corresponding correlations between the immune checkpoint genes and risk scores are shown in Supplementary Figure 9.

The above results suggest that the ability of TMB to predict the response to immunotherapy in HNSCC patients is related to the target genes of immunotherapy. Furthermore, the use of various immune checkpoint inhibitors for HNSCC patients with different risks based on differences in immune infiltration and immune checkpoint genes may be more beneficial to patients.

### Exploration of Drug Sensitivity Based on the Prognostic Model

**FIGURE 7:**
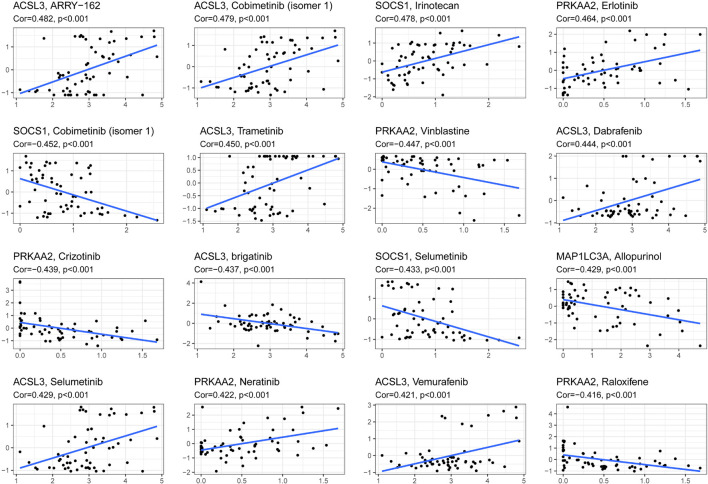
The correlation between ferroptosis-related genes to construct the prognostic model and drug sensitivity.

The reliability of the risk score prognostic model and nomogram based on the model was validated by multiple methods, and it is necessary to reduce the risk of HNSCC. Figure 7 exhibits that, in the representative 16 correction analysis, ACSL3 expression is positively correlated with the sensitivity of cancer patients to ARRY-162 (MEK162), and SOCS1 expression is negatively correlated with the sensitivity of cancer patients to cobimetinib (isomer 1), thereby promoting novel research on targeted drugs.

## Discussion

Excluding communicable diseases, approximately 30% of premature deaths are attributable to cancer in adults aged approximately 30–69 years (WHO, 2020). As a malignant tumor with increasing incidence, the OS of HNSCC patients is unsatisfactory with the currently available treatments, and HNSCC recurrence with a 50% ratio in locally advanced disease further reduces the 5-year survival rate (Muzaffar et al., 2021). Therefore, identifying HNSCC patients with poor prognosis and developing more effective treatments to promote individualized treatment and improve clinical outcomes are urgently required.

Ferroptotic cell death, a relatively novel cell death mechanism, has been shown to improve the curative effect of radiotherapy and immunotherapy in cancer (Lang et al., 2019). However, few studies have investigated the relationship between ferroptosis-related genes and OS in HNSCC patients. In this study, a novel prognostic risk model for HNSCC was first built based on ferroptosis-related genes, and a clinical prognostic model was constructed to assess patient prognosis.

In the present study, we systematically investigated the correlation between ferroptosis-related genes and HNSCC. A total of 175 differentially expressed ferroptosis-related genes were identified in HNSCC based on TCGA, GTEx, FerrDb, and MSigDB, which provided a reliable foundation for future exploration. The 10 independent prognostic genes were screened to construct the risk score model based on univariate, LASSO, and multivariable logistic regression algorithms, which avoided overfitting and improved the clinical practicability of the model.

In the 10 ferroptosis-related genes, the coefficients of MAP1LC3A, MAP3K5, and SOCS1 were negatively correlated with the prognostic risk score, suggesting that these 3 genes might induce ferroptosis in HNSCC. However, the coefficients of the other 7 genes were positively correlated with the prognostic risk score, suggesting that these 7 genes might inhibit ferroptosis in HNSCC. Moreover, the potential gene functions and pathways of these 10 genes were further explored. Several results have been suggested to be correlated with ferroptosis in the top 10 KEGG and GO enrichment analyses. The top 10 results of GO analysis were primarily enriched in biological processes related to cellular responses to chemical stress, starvation, nutrient levels, and oxidative stress. Previous studies suggested that chemotherapeutic agents, such as sulfasalazine and cisplatin, might induce ferroptosis by acting on system xc- or GSH, which play a key role in ferroptosis development (Mou et al., 2019). Oxidative stress, as an important characteristic, has been identified as a crucial factor in ferroptosis induction (Mou et al., 2019). Moreover, the top 10 gene functions in the GO analysis are related to the cell response to nutrients and starvation, suggesting that deregulating cellular energetics might participate in ferroptosis. Energy stress has been reported to inhibit lipid peroxidation and ferroptosis (Lee et al., 2020). For the KEGG enrichment analysis, the top result was ferroptosis, which further suggested that the 10 genes might participate in ferroptosis and HNSCC development. Autophagy may promote ferroptotic death by ferritinophagy, lipophagy, or clockophagy (Liu et al., 2020). Moreover, cell sensitivity to ferroptosis might be affected by mTOR pathway regulation (Yi et al., 2020). In summary, the most significant gene functions and pathways from GO and KEGG analyses indicated that the mechanism of the 10 genes might be correlated with ferroptosis.

The majority of the 10 signature genes in the prognostic model have been shown to be involved in multiple cancers. However, the role of several genes in HNSCC prognosis remains to be explored. Ovarian tumor domain-containing ubiquitin aldehyde binding protein 1 (OTUB1) is an important deubiquitinating enzyme (DUB) that inhibits the ubiquitination of E2s following cleavage of the ubiquitin chains in target proteins (Wiener et al., 2012). OTUB1 has complex functions in various cancers, promotes tumor migration, and is a tumor suppressor that induces cell apoptosis and cell growth by regulating the DNA damage response (Sun et al., 2012; Herhaus et al., 2013; Kato et al., 2014). Moreover, OTUB1 may influence immune factor production. OTUB1 deletion downregulates the synthesis of the protective chemokine INF-γ, which is secreted by NK cells (Mulas et al., 2021). In addition, OTUB1 participates in metabolic reprogramming, which is an essential mechanism for activated T cell function (Pearce et al., 2013). Acyl-coenzyme A (CoA) synthetase long-chain family member 3 (ACSL3) regulates fatty acid metabolism and is a substrate for both β-oxidation and lipid synthesis after converting free long-chain fatty acids into fatty acyl-CoA esters (Coleman et al., 2002). Lipid metabolic reprogramming is one of the most prominent metabolic changes in cancer cells and has received increasing attention. As an important component of lipid metabolism, fatty acid deregulation might disturb the curative effect of radiotherapy and chemotherapy and affect immunotherapy for cancer patients (Wu et al., 2009; Han et al., 2019; Corn et al., 2020). ACSL enzymes, including ACSL3, have been suggested to induce apoptosis in a subset of TP53-deficient cancer cells (Yamashita et al., 2000; Mashima et al., 2005). Moreover, ACSL3 has been shown to be essential for mutant KRAS lung cancer tumorigenesis (Padanad et al., 2016). Mitogen-activated protein kinase 5 (MAP3K5) has been suggested to be involved in multiple biological activities, including stress-induced apoptosis and cell differentiation (Subramanian et al., 2004; Mizumura et al., 2006). As an important apoptosis signal-regulating kinase, MAP3K5 catalytic activity leads to differentially regulated apoptosis by inducing DNA damage, ROS, and tumor necrosis factor (Nagai et al., 2007). Moreover, MAP3K5 has been shown to play an important role in innate immune responses through the production of proinflammatory cytokine production (Matsuzawa et al., 2005). In general, ferroptosis-related genes are correlated with the human immune system, prompting subsequent analysis of immune function based on the present model.

After the model was constructed, K-M analysis and ROC curves were used to determine the effect of the model to identify HNSCC patients at different risks, and the test method revealed a satisfactory effect in predicting HNSCC patient prognosis.

The K-M analysis of our model showed a more optimistic trend; the difference in survival curve in the high- and low-risk groups was more significant compared with that of the previous study (He et al., 2021). Moreover, the prognostic risk model of HNSCC patients was more accurate than the models reported in certain previous studies with a preferable AUC for 1 (0.665 vs. 0.634 and 0.663), 3 (0.743 vs. 0.672 and 0.686), and 5 years (0.755 vs. 0.642 and 0.622) (Yang et al., 2020; Liu B. et al., 2021). Interestingly, the AUC values of our model increased annually, which further strengthened the prognostic effect of the model. Moreover, the internal validation in the test cohort and external validation in the entire cohort and the MTAB-8588 dataset further improved model stringency. Overall, the practicability of the model was validated using multiple methods.

To further explore the clinical value of the model in HNSCC patients, the risk score was integrated with the clinical parameters of HNSCC patients. The risk score based on the model was an independent prognostic parameter that had synergistic effects with other independent prognostic characteristics to further improve the value of the nomogram to calculate individual prognoses, which were validated by the C-index, ROC curve, calibration plot, and DCA. The nomogram designed to predict HNSCC patient prognosis was more accurate than the models reported in a previous study with a superior C-index (0.752 vs. 0.640) and AUC for 1 (0.729 vs. 0.597), 3 (0.828 vs. 0.706), and 5 years (0.853 vs. 0.645) (He et al., 2021). Surprisingly, the AUC values of the nomogram at 1, 3, and 5 years increased annually, and the trend was consistent with the AUC values of the risk score model, which further strengthened the reliability of the risk score model and nomogram. Moreover, internal and external validation revealed optimistic effects for the nomogram.

Recently, tumor immunotherapy has been considered a breakthrough in oncotherapy. However, the response to immunotherapy differs between cancers and within cohorts with the same cancer (Ventola, 2017). Thus, the TMB level was developed to predict the response to immunotherapy in cancer patients, and high TMB has been shown to be positively correlated with the curative effect of immunotherapy (Galuppini et al., 2019). However, it has been reported that a high TMB fails to predict the immune checkpoint blockade response across all cancers (McGrail et al., 2021).

After analyzing the correlation between the risk score and TMB in HNSCC patients, immune cell infiltration in the tumor microenvironment and the differences in immune checkpoints of high- and low-risk HNSCC patients were further investigated to explore the curative effect of immunotherapy based on the risk score model. The relationship between ferroptosis and the immune microenvironment is complicated. Cancer cells with ferroptosis can increase immunogenicity by releasing immune-stimulating signals, which allow immune cells, such as macrophages and dendritic cells (DCs), to locate the cancer cell site (Friedmann Angeli et al., 2019; Tang et al., 2021). Early ferroptotic cancer cells can elicit a vaccination-like effect by promoting phenotypic DC maturation (Efimova et al., 2020). The increased immunogenicity of cancer cells may enhance the efficacy of immune checkpoint inhibitors. Moreover, immune cells, such as CD8 + cells, can trigger lipid peroxidation and cause ferroptosis in tumor cells, which suggests that immune checkpoint inhibitors targeting CD8 + cells might act synergistically with ferroptosis (Wang et al., 2019). In addition to predicting HNSCC patient prognosis, differences in the immune microenvironment based on the prognostic risk model might be beneficial for developing individualized immunotherapy and improving the curative effect of HNSCC patients.

Moreover, the analysis of drug sensitivity based on the model suggested the possibility of targeting the 10 gene risk signatures and reducing the prognostic risk of HNSCC patients. The drugs might regulate the ferroptosis level of cancer cells by targeting the 10 ferroptosis-related genes. ACSL3 was found to contribute the most to drug sensitivity. ARRY-162 (MEK162), as the drug with the greatest correlation with ACSL3, is a highly selective MEK1/2 inhibitor that could offer a new combination therapy with ferroptosis and immunotherapy (Erkes et al., 2020; Gagliardi et al., 2020).

The present study has several limitations. Certain *in vitro* and *in vivo* experiments, such as the in-depth molecular mechanisms of the ferroptosis-related genes, needed to construct the prognostic model require further verification in experimental studies. Moreover, the study was based only on research data from public databases, which might have contributed to the selection bias. Thus, a multicenter and large-scale study is required to further validate the clinical utility of our model.

## Conclusion

In summary, the present study systematically investigated the correlation between ferroptosis-related genes and HNSCC patient prognosis, and a novel prognostic model of HNSCC patients based on 10 ferroptosis-related genes was established and validated. Furthermore, the values of the model to be used in predicting prognosis with other clinical parameters, immunotherapy, and drug sensitivity of HNSCC patients were positive, indicating that the novel ferroptosis-related gene signatures might be beneficial in developing individualized treatments, thereby improving the OS of HNSCC patients.

## Data Availability Statement

The datasets used and analysed for this study were obtained from TCGA 487 (https://portal.gdc.cancer.gov/) and ArrayExpress database 488 (https://www.ebi.ac.uk/arrayexpress/).

## Author Contributions

DH and WH: conceptualization and project administration. SL and LX: methodology and investigation. SL, LX, LH, and LC: formal analysis. DH: writing – original draft preparation. WH: writing – review and editing and supervision. DH, LH, and MY: visualization. MY and SL: funding acquisition. All authors support publishing, contributed to the article, and approved the submitted version.

## Conflict of Interest

The authors declare that the research was conducted in the absence of any commercial or financial relationships that could be construed as a potential conflict of interest.

## Publisher’s Note

All claims expressed in this article are solely those of the authors and do not necessarily represent those of their affiliated organizations, or those of the publisher, the editors and the reviewers. Any product that may be evaluated in this article, or claim that may be made by its manufacturer, is not guaranteed or endorsed by the publisher.

## Footnotes

1
https://portal.gdc.cancer.gov/

2
https://gtexportal.org/home/

3
https://www.ebi.ac.uk/arrayexpress/

4
https://discover.nci.nih.gov/cellminer/

## Supplementary Material

Supplementary Figure 1 Multivariable Cox regression analysis to construct a prognostic risk score model.

Supplementary Figure 2 K-M survival analysis and ROC curves of risk prognostic model in internal and external validation. **(A–C)** K-M survival analysis of risk prognostic model of HNSCC patients in internal, entire, and external validated cohorts. **(D–L)** ROC curves analysis of risk prognostic model of HNSCC patients at 1, 3, and 5 years in internal, entire, and external validated cohorts.

Supplementary Figure 3 Representative results of GO and KEGG analyses. **(A)** The GO analysis of the 10 screened genes. **(B)** The potential signaling pathway of the 10 screened genes.

Supplementary Figure 4 Independent prognostic parameter identification in HNSCC patients. **(A)** The univariate Cox regression analysis of clinical parameters in HNSCC patients. **(B)** The multivariate Cox regression analysis of clinical parameters in HNSCC patients.

Supplementary Figure 5 The validation of nomogram in internal and external cohorts. **(A–C)** ROC curves to assess nomogram accuracy to predict 1-, 3-, and 5-year OS in internal cohort. **(D–F)** ROC curves to assess nomogram accuracy to predict 1-, 3-, and 5-year OS in entire cohort. **(G–I)** ROC curves to assess nomogram accuracy to predict 1-, 3-, and 5-year OS in external cohort.

Supplementary Figure 6 The analysis between risk score and different clinical parameters in HNSCC patients. **(A)** The difference of risk score is significant in HNSCC patients with different stage. **(B)** The difference of risk score is significant in HNSCC patients with different N stage. **(C)** The difference of risk score is significant in HNSCC patients with different lymphovascular invasion. **(D)** The difference of risk score is significant in HNSCC patients with different perineural invasion.

Supplementary Figure 7 The different immune infiltration in the high- and low-risk HNSCC patients. **(A,B)** The expression of eight immune cell types is higher in the low-risk group compared with that of the high-risk group. **(C)** The expression of four cell types is higher in the high-risk group compared with that of the low-risk group.

Supplementary Figure 8 The different immune checkpoint genes in the high- and low-risk HNSCC patients. **(A,B)** The expression of 10 immune checkpoint gene types is higher in the low-risk group compared with that of the high-risk group. **(C)** The expression of two immune checkpoint gene types is higher in the high-risk compared with that of the low-risk group.

Supplementary Figure 9 The correlations between the immune checkpoint genes and risk score. **(A–J)** The immune checkpoint genes were negatively correlated with the risk score in HNSCC patients. **(K,L)** The immune checkpoint genes were positively correlated with the risk score in HNSCC patients.

Supplementary Table 1 Univariate COX regression analysis of differentially expressed ferroptosis-related genes in HNSCC patients.
