# Supplementary figures and images for: Prognostic Value of a Ferroptosis-Related Gene Signature in Patients With Head and Neck Squamous Cell Carcinoma
Source: Front Cell Dev Biol. 2021 Nov 1;9:739011. doi: 10.3389/fcell.2021.739011 (PMC8591309; doi:10.3389/fcell.2021.739011)

# Hazard ratio

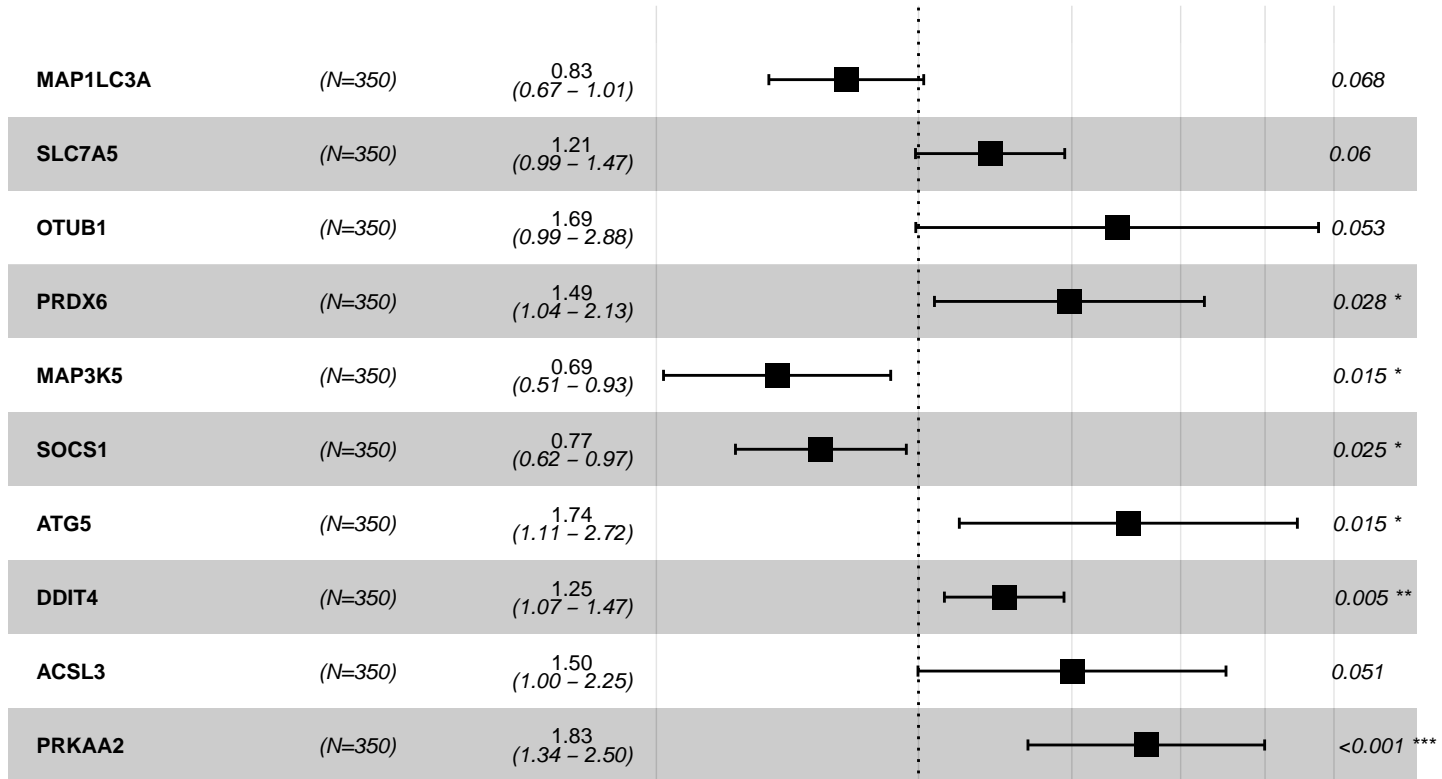

# Events: 138; Global p-value (Log-Rank): 2.877e-09

AIC: 1350.21; Concordance Index: 0.67

0.5

1

1.5

2

2.5

3

Supplement: Supplementary Figure 1 — Multivariable Cox regression analysis to construct a prognostic risk score model. [file Data_Sheet_2.PDF]

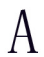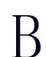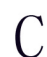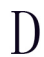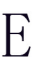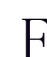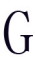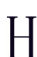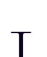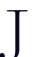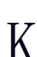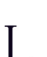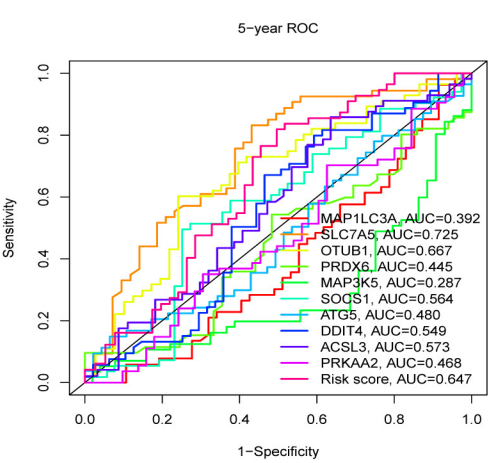

Supplement: Supplementary Figure 2 — K-M survival analysis and ROC curves of risk prognostic model in internal and external validation. (A–C) K-M survival analysis of risk prognostic model of HNSCC patients in internal, entire, and external validated cohorts. (D–L) ROC curves analysis of risk prognostic model of HNSCC patients at 1, 3, and 5 years in internal, entire, and external validated cohorts. [file Data_Sheet_3.PDF]

A

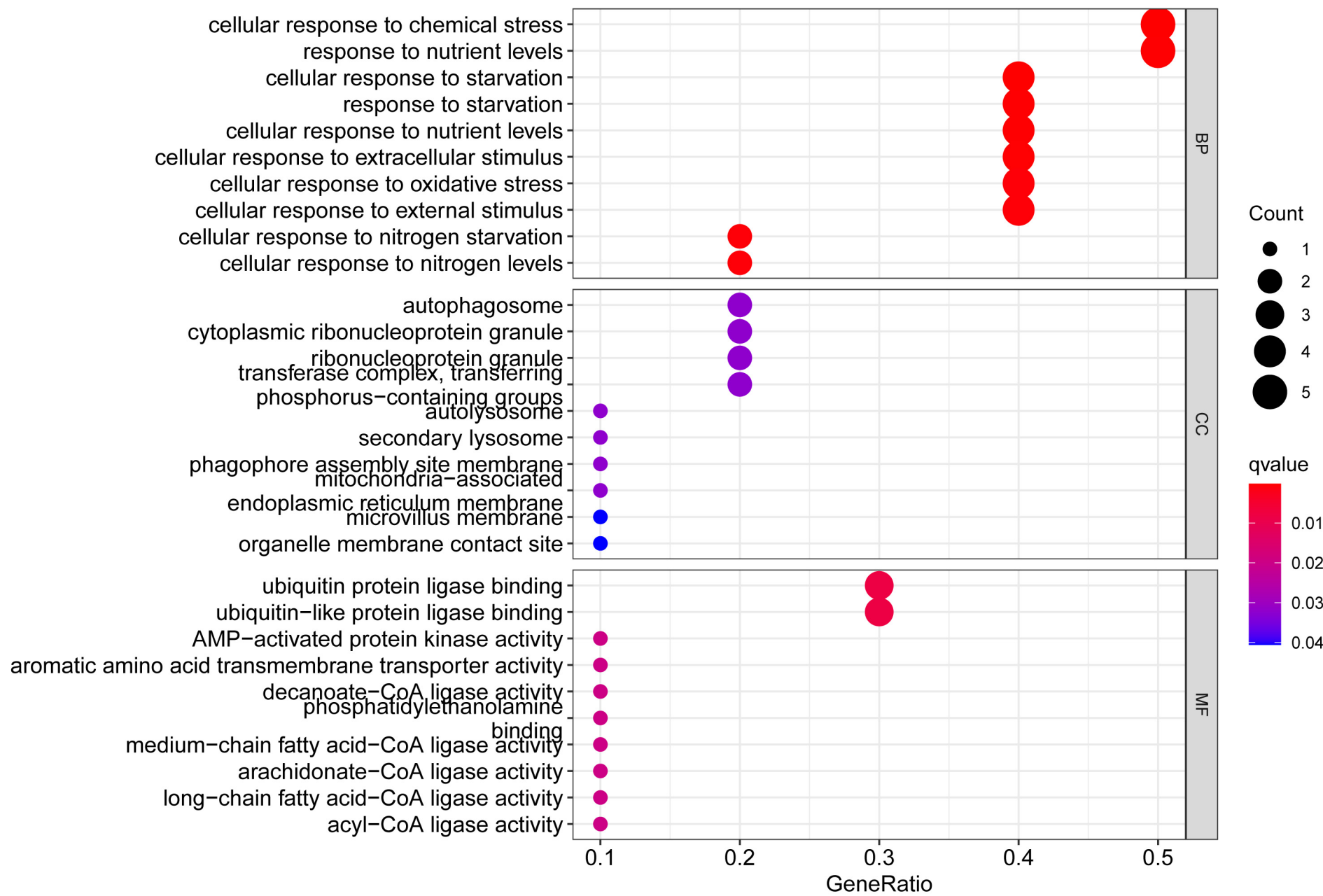

B

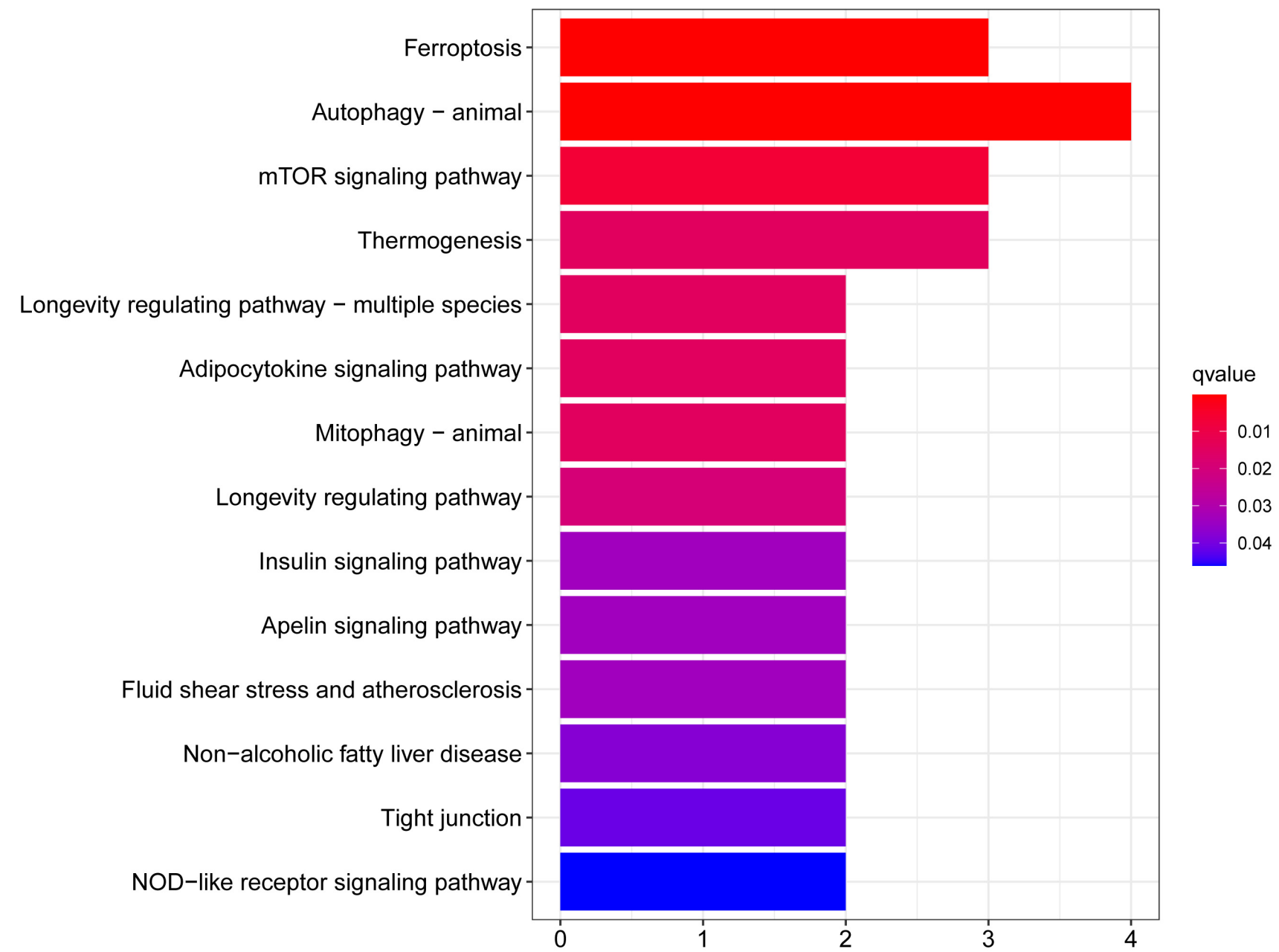

Supplement: Supplementary Figure 3 — Representative results of GO and KEGG analyses. (A) The GO analysis of the 10 screened genes. (B) The potential signaling pathway of the 10 screened genes. [file Data_Sheet_4.PDF]

A

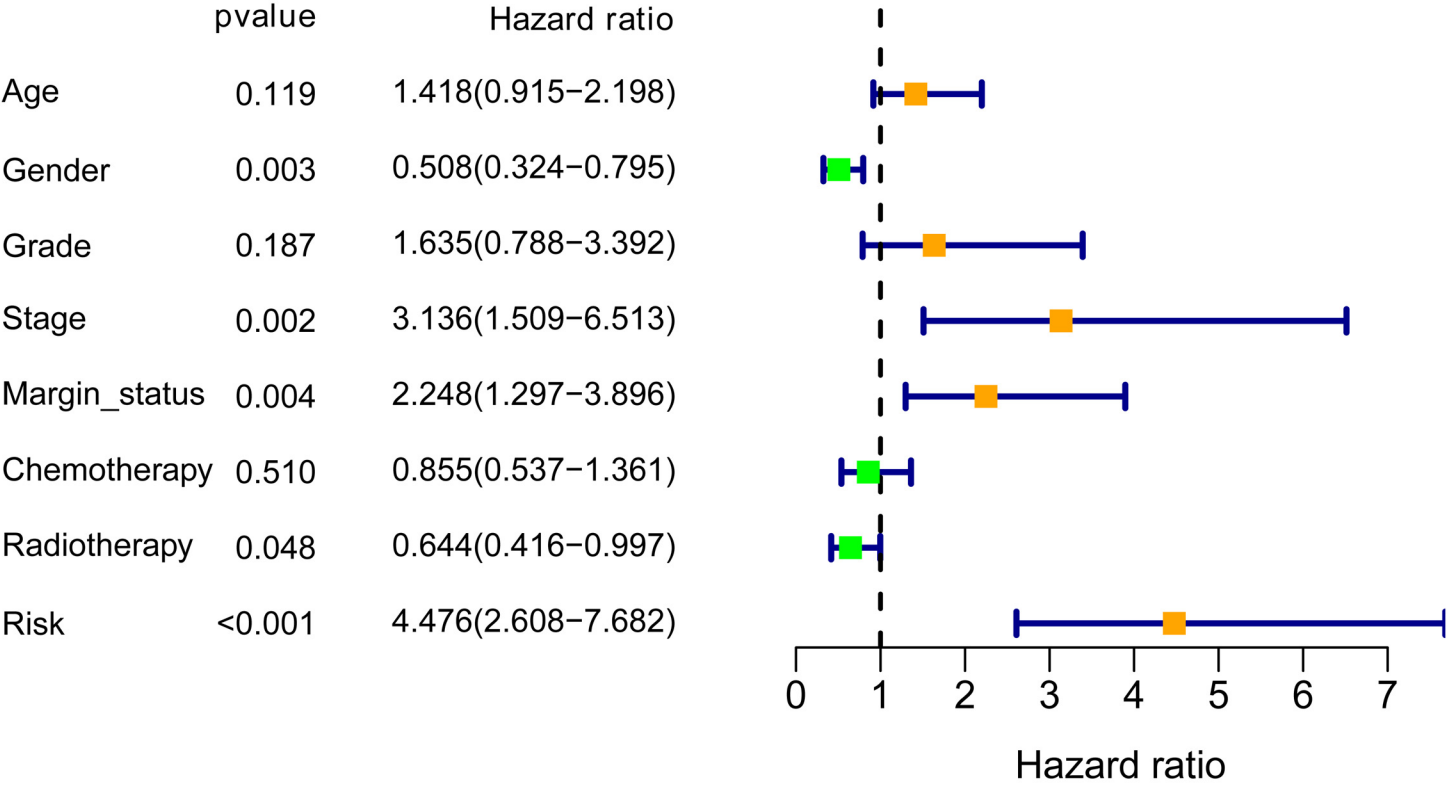

B

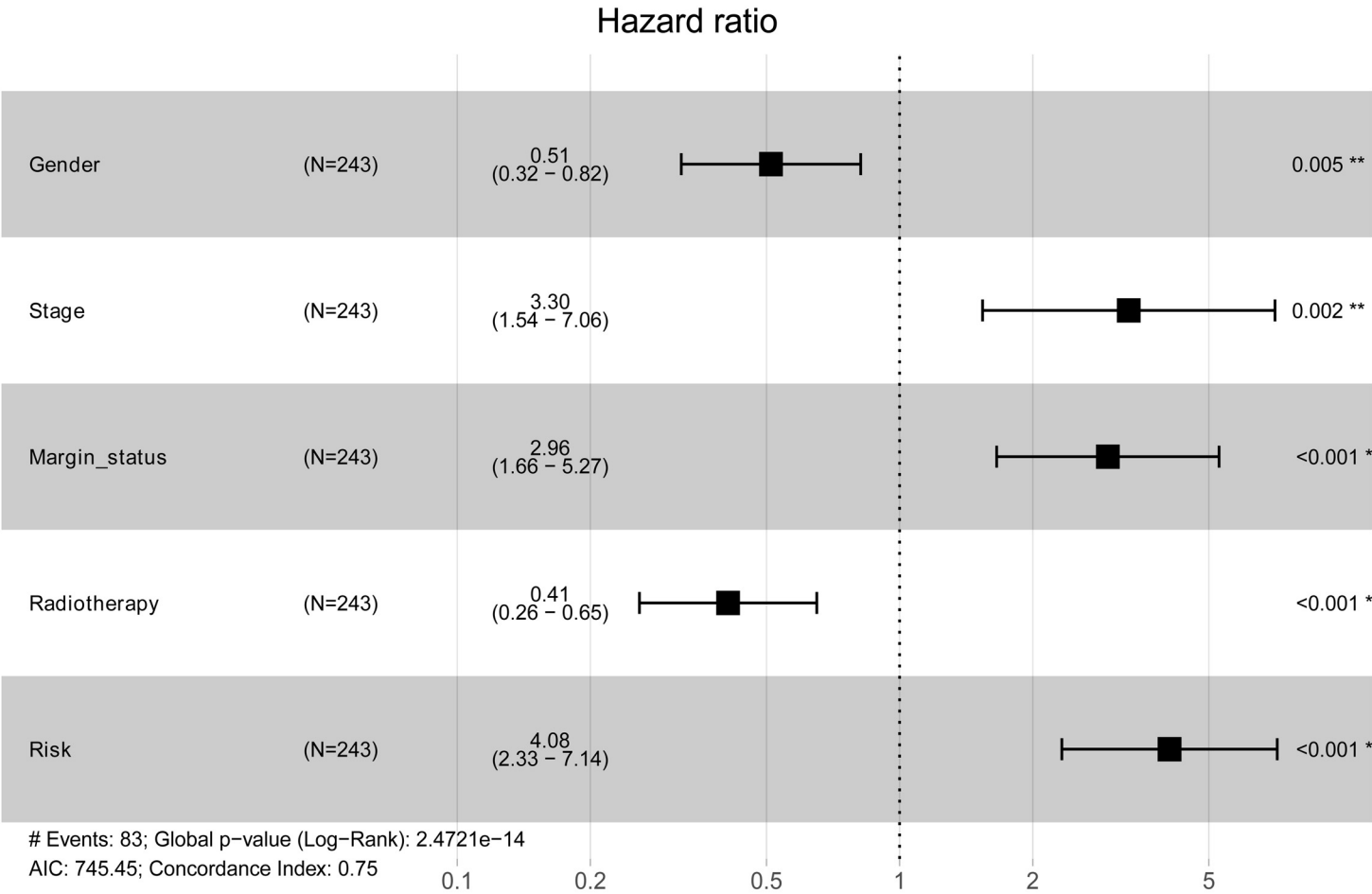

Supplement: Supplementary Figure 4 — Independent prognostic parameter identification in HNSCC patients. (A) The univariate Cox regression analysis of clinical parameters in HNSCC patients. (B) The multivariate Cox regression analysis of clinical parameters in HNSCC patients. [file Data_Sheet_5.PDF]

A

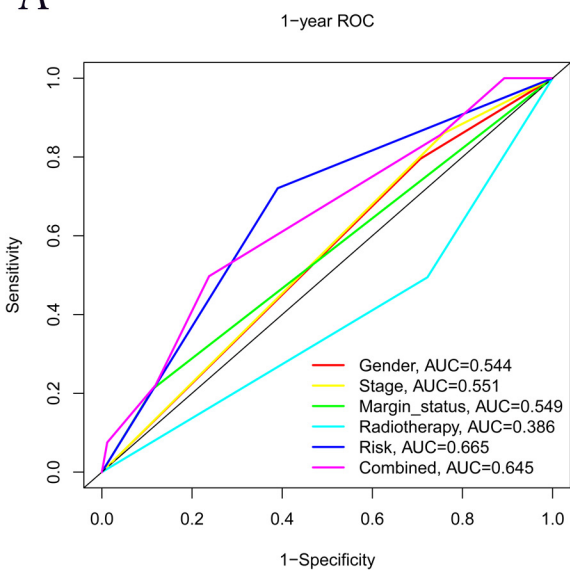

B

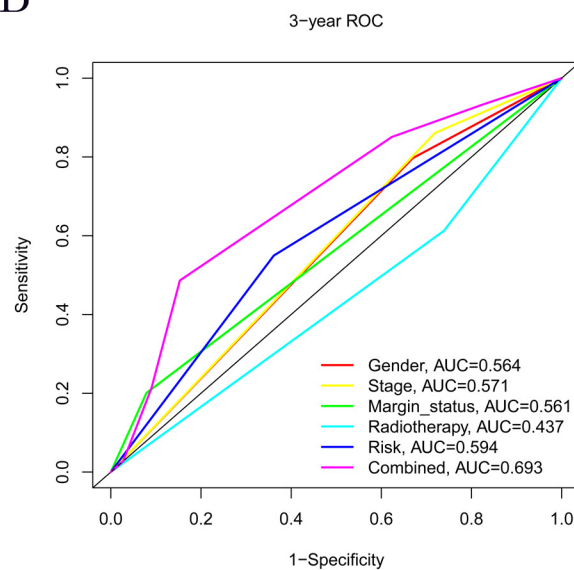

C

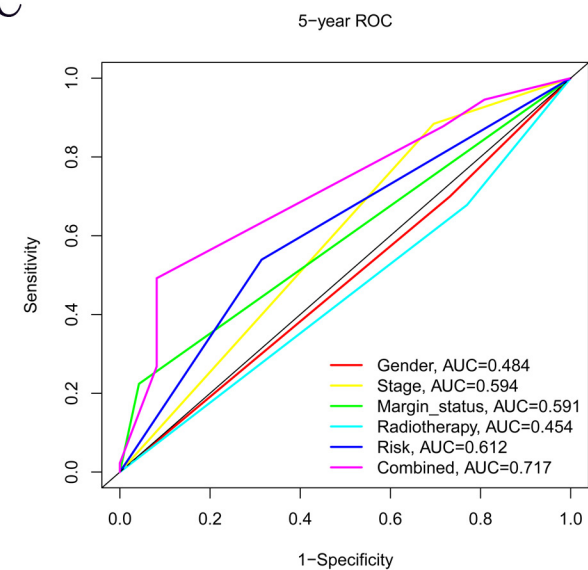

D

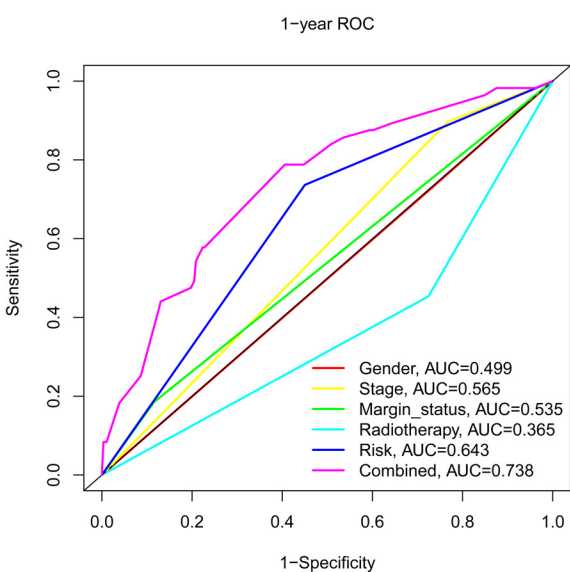

E

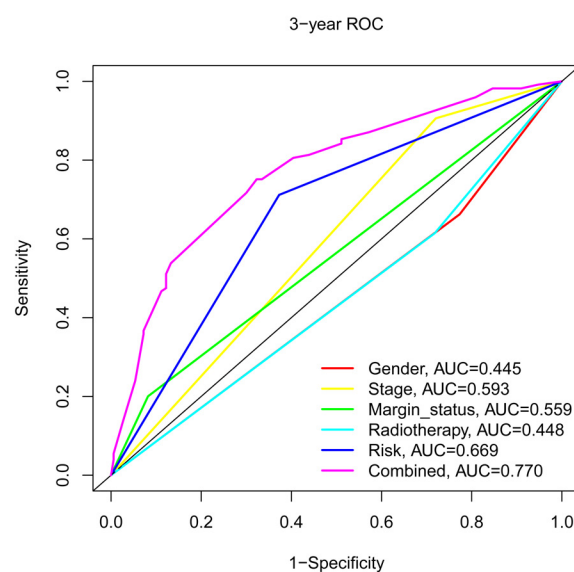

F

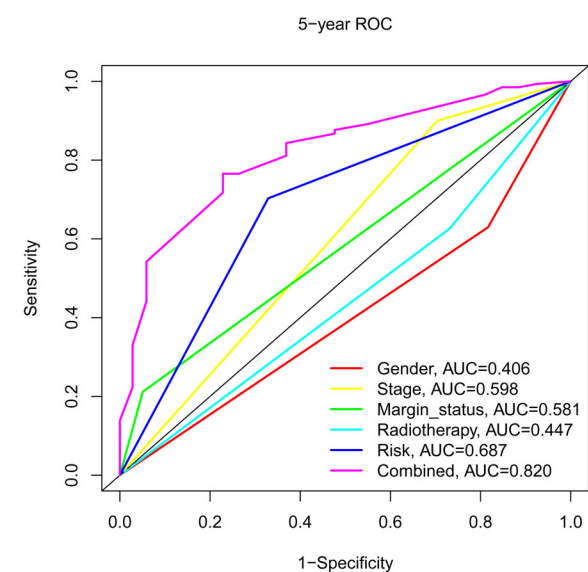

G

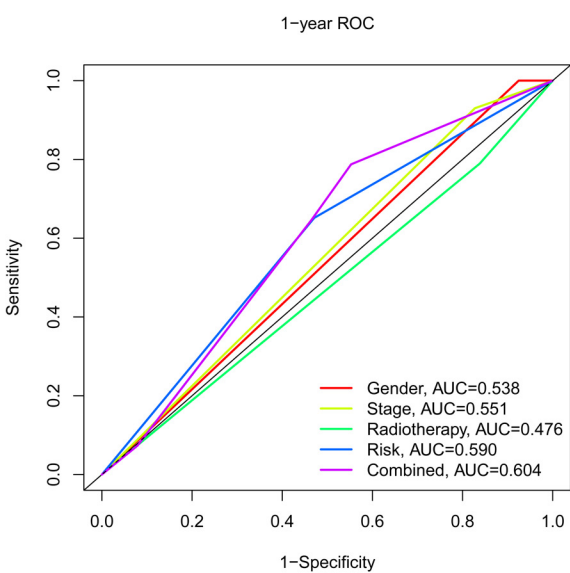

H

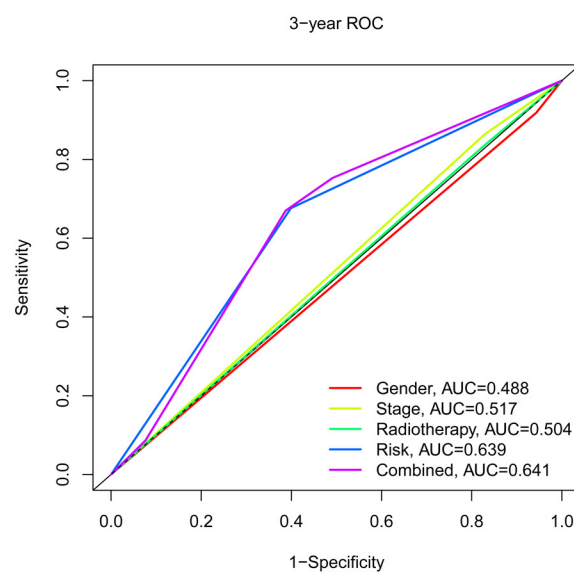

I

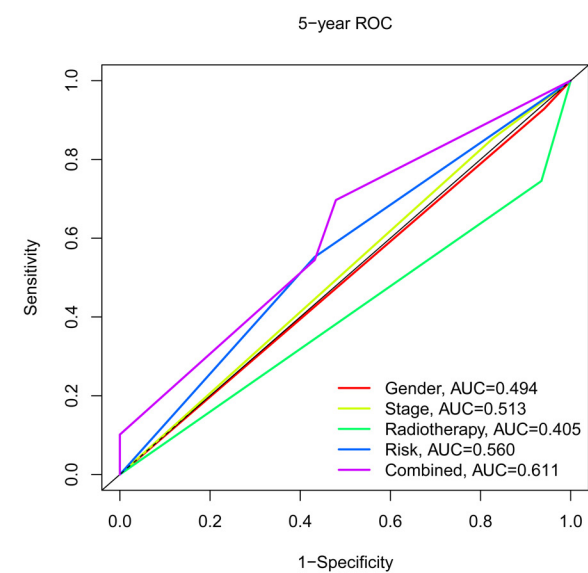

Supplement: Supplementary Figure 5 — The validation of nomogram in internal and external cohorts. (A–C) ROC curves to assess nomogram accuracy to predict 1-, 3-, and 5-year OS in internal cohort. (D–F) ROC curves to assess nomogram accuracy to predict 1-, 3-, and 5-year OS in entire cohort. (G–I) ROC curves to assess nomogram accuracy to predict 1-, 3-, and 5-year OS in external cohort. [file Data_Sheet_6.PDF]

A

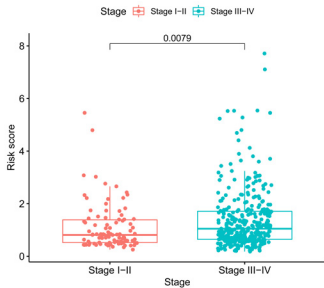

B

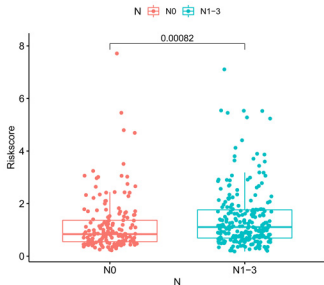

C

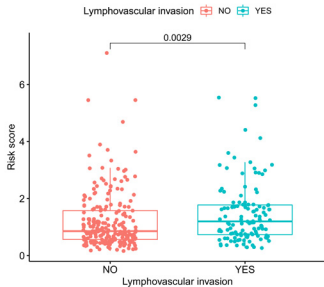

D

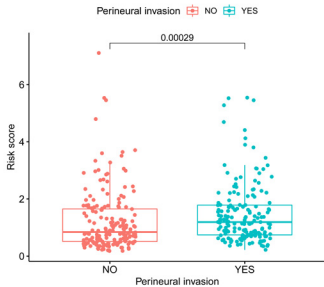

Supplement: Supplementary Figure 6 — The analysis between risk score and different clinical parameters in HNSCC patients. (A) The difference of risk score is significant in HNSCC patients with different stage. (B) The difference of risk score is significant in HNSCC patients with different N stage. (C) The difference of risk score is significant in HNSCC patients with different lymphovascular invasion. (D) The difference of risk score is significant in HNSCC patients with different perineural invasion. [file Data_Sheet_7.PDF]

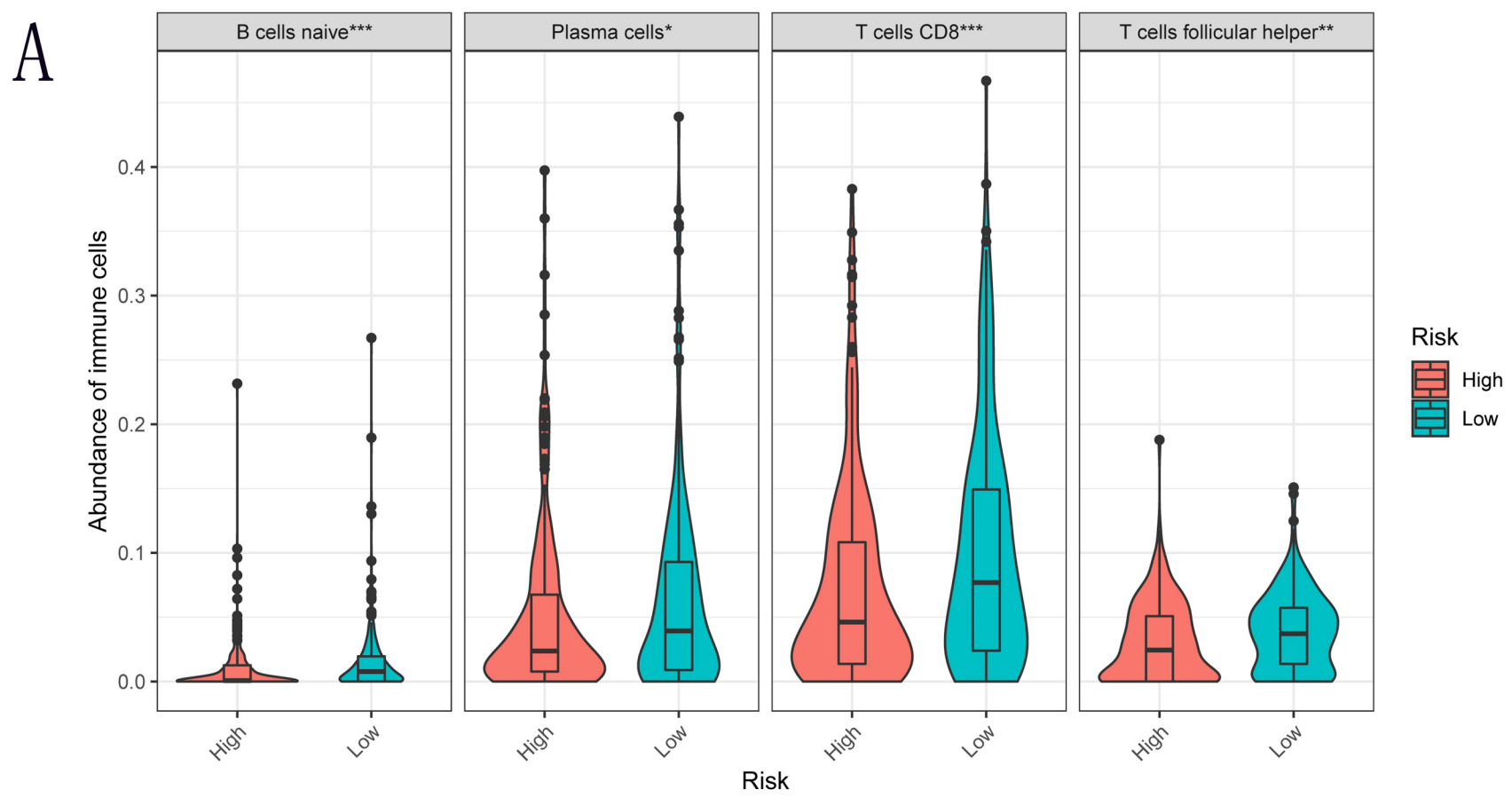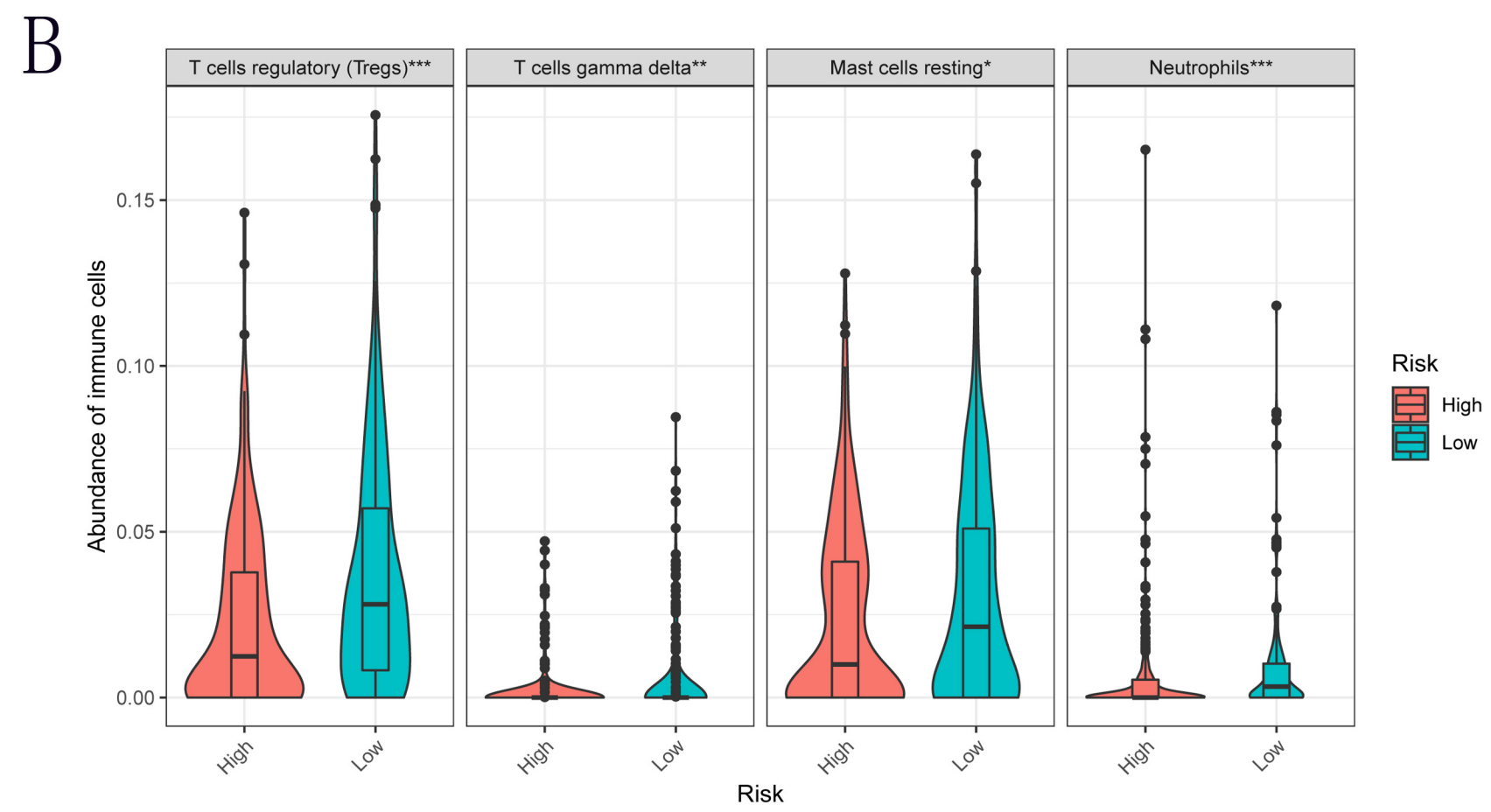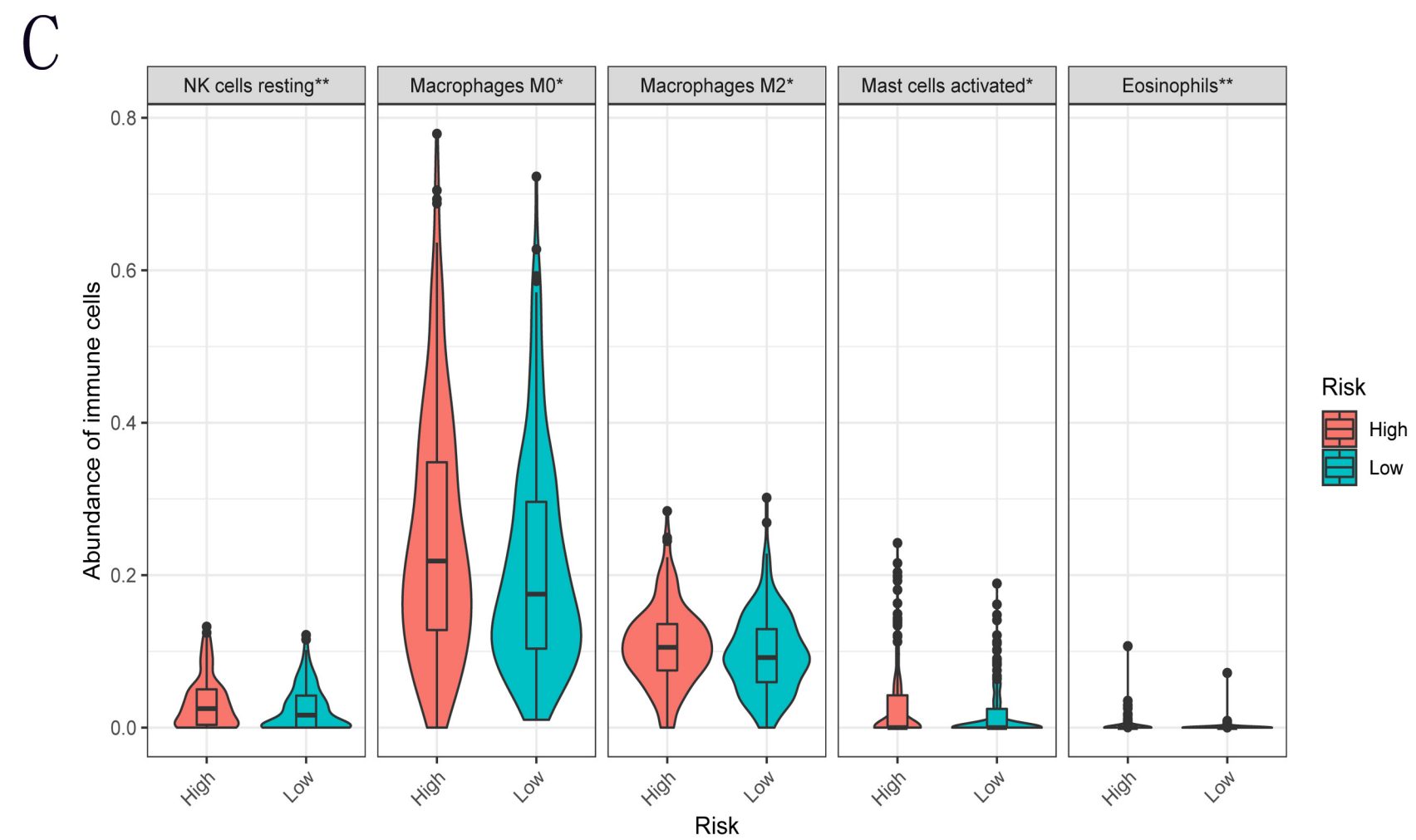

Supplement: Supplementary Figure 7 — The different immune infiltration in the high- and low-risk HNSCC patients. (A,B) The expression of eight immune cell types is higher in the low-risk group compared with that of the high-risk group. (C) The expression of four cell types is higher in the high-risk group compared with that of the low-risk group. [file Data_Sheet_8.PDF]

A

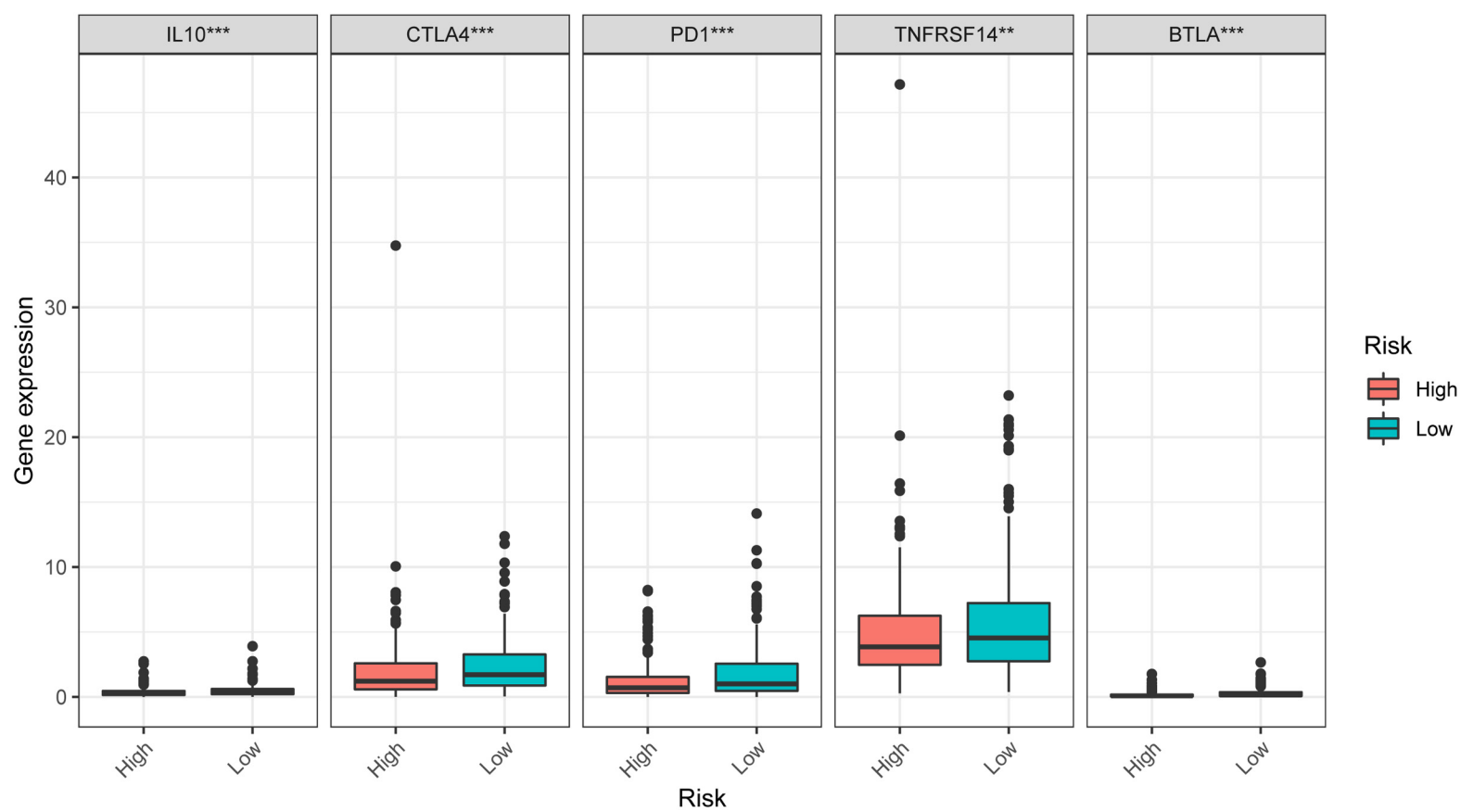

B

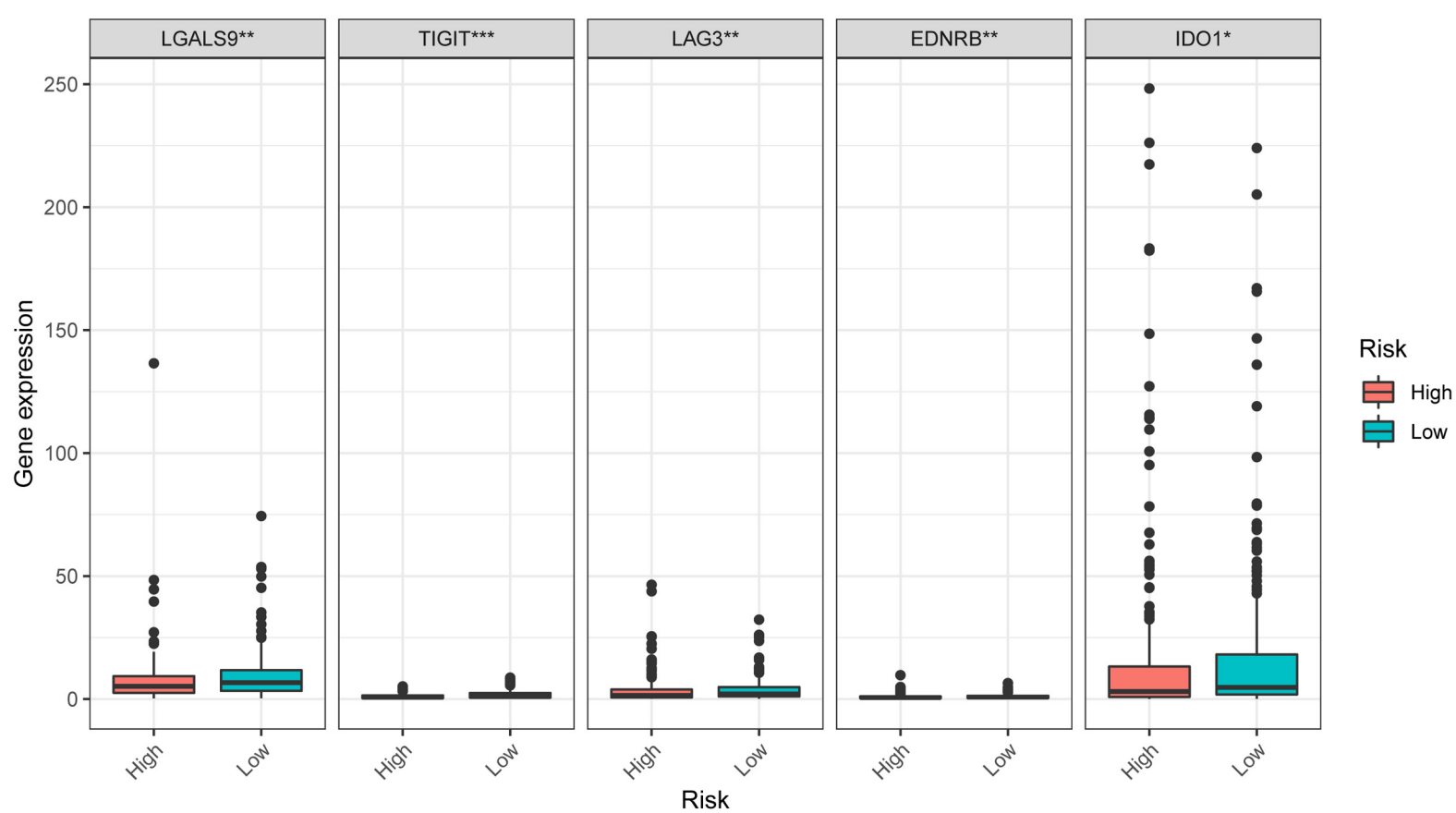

C

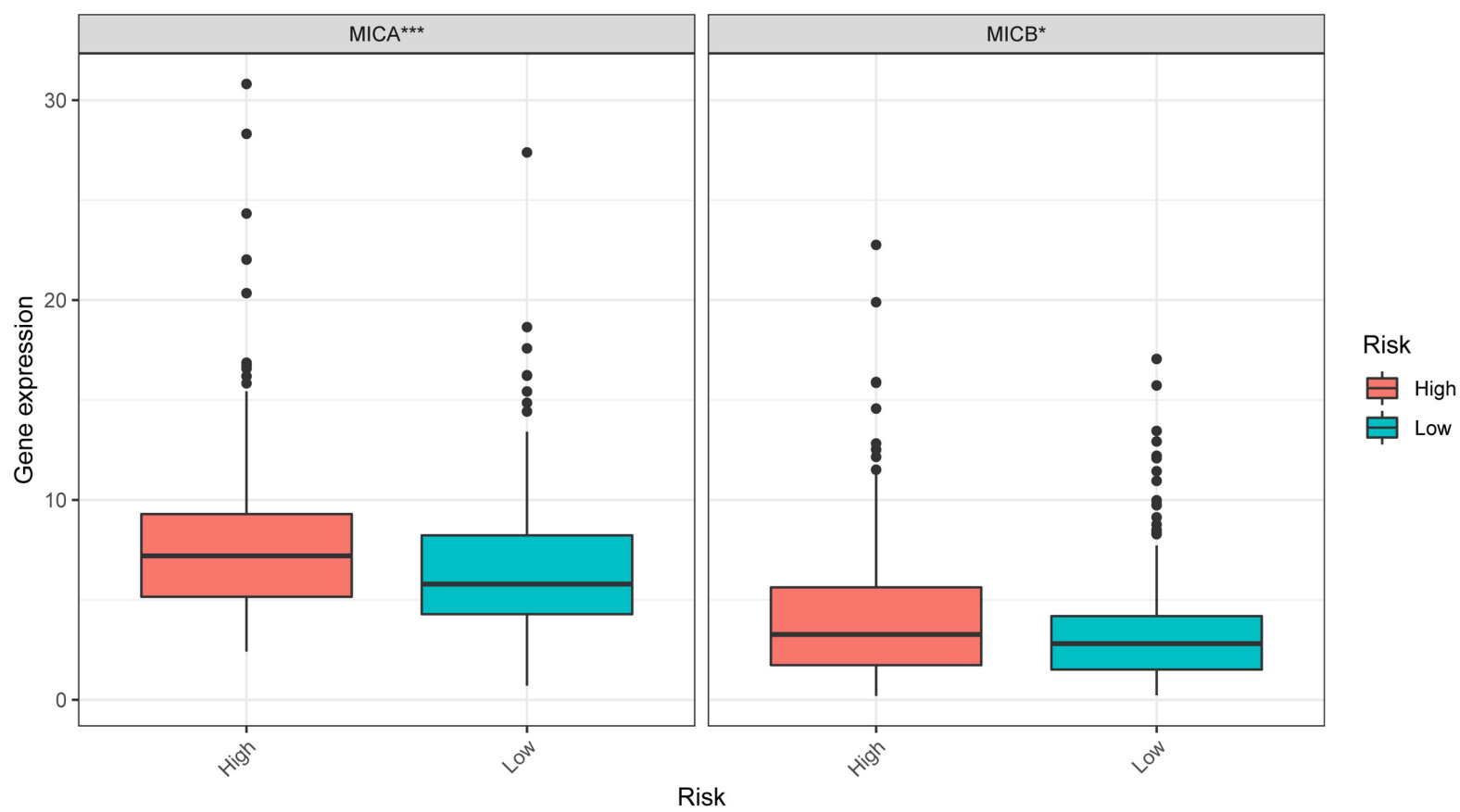

Supplement: Supplementary Figure 8 — The different immune checkpoint genes in the high- and low-risk HNSCC patients. (A,B) The expression of 10 immune checkpoint gene types is higher in the low-risk group compared with that of the high-risk group. (C) The expression of two immune checkpoint gene types is higher in the high-risk compared with that of the low-risk group. [file Data_Sheet_9.PDF]

A

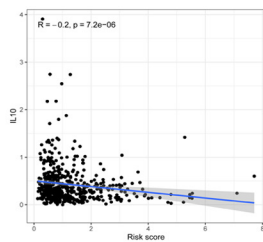

B

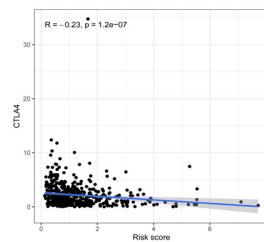

C

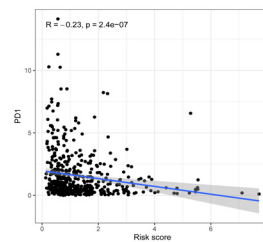

D

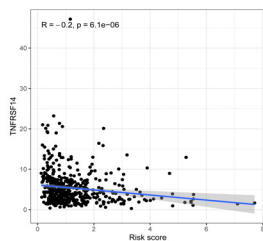

E

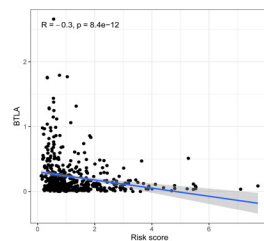

F

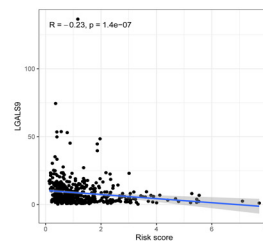

G

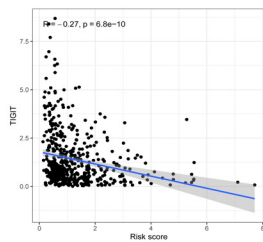

H

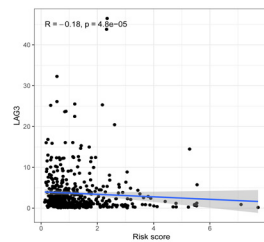

I

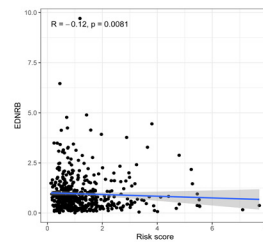

J

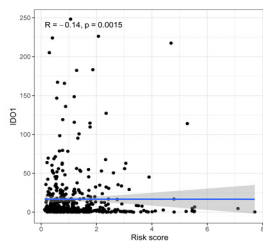

K

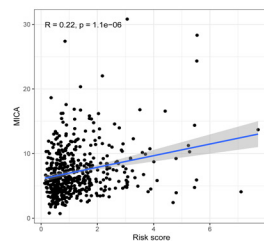

L

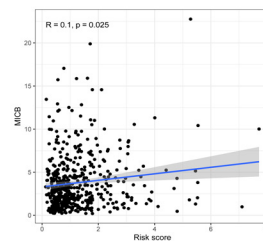

Supplement: Supplementary Figure 9 — The correlations between the immune checkpoint genes and risk score. (A–J) The immune checkpoint genes were negatively correlated with the risk score in HNSCC patients. (K,L) The immune checkpoint genes were positively correlated with the risk score in HNSCC patients. [file Data_Sheet_10.PDF]
