# Supplementary material for: Prognostic Value of a Ferroptosis-Related Gene Signature in Patients With Head and Neck Squamous Cell Carcinoma
Source: Front Cell Dev Biol. 2021 Nov 1;9:739011. doi: 10.3389/fcell.2021.739011 (PMC8591309; doi:10.3389/fcell.2021.739011)
Supplement: Supplementary Table 1 — Univariate COX regression analysis of differentially expressed ferroptosis-related genes in HNSCC patients. [file Table_1.DOCX]

**Table S1** Univariate COX regression analyses of differentially

expressed ferroptosis-related genes in HNSCC patients

Genes univariate analysis P value

HR(95%CI)

TRIB3 1.469(1.178-1.831) <0.001

PRKAA2 1.466(1.122-1.914) <0.01

NQO1 1.191(1.052-1.349) <0.01

PRDX6 1.554(1.126-2.146) <0.01

SLC7A5 1.254(1.061-1.481) <0.01

MAP3K5 0.705(0.539-0.922) 0.011

GABARAPL2 1.735(1.127-2.670) 0.012

FTH1 1.371(1.069-1.758) 0.013

OTUB1 1.753(1.125-2.730) 0.013

HSPA5 1.536(1.082-2.181) 0.016

VDAC2 1.611(1.078-2.406) 0.020

YWHAE 1.735(1.090-2.764) 0.020

ACSL3 1.608(1.074-2.407) 0.021

DDIT4 1.197(1.026-1.397) 0.022

SOCS1 0.795(0.649-0.973) 0.026

ATG5 1.650(1.060-2.566) 0.026

MAP1LC3A 0.812(0.673-0.979) 0.029

FBXW7 0.593(0.370-0.951) 0.030

TXNRD1 1.154(1.012-1.315) 0.032

CAV1 1.149(1.008-1.310) 0.037

MAP1LC3B 1.616(1.028-2.541) 0.038

SLC3A2 1.301(1.015-1.667) 0.038

GABARAPL1 1.245(1.006-1.542) 0.044

SLC7A11 1.133(1.003-1.280) 0.045

RIPK1 0.639(0.409-0.999) 0.049
